# Adherence to the United States Department of Agriculture Dietary Recommendations Pre- and During the Coronavirus Disease-19 Pandemic Among Pregnant Women in Arab Countries

**DOI:** 10.3389/fnut.2022.824305

**Published:** 2022-03-17

**Authors:** Maha Hoteit, Reem Hoteit, Ayoub Al-Jawaldeh, Mariane Abou Nasr, Sara Obeid, Chadi Fakih, Mohamad El Hajj, Radwan Qasrawi, Rania Abu Seir, Sabika Allehdan, Mahmoud Samy Ismail, Khlood Bookari, Jamila Arrish, Nahla Al-Bayyari, Reema Tayyem

**Affiliations:** ^1^Faculty of Public Health, Lebanese University, Beirut, Lebanon; ^2^PHENOL Research Group Public Health Nutrition Program-Lebanon, Faculty of Public Health, Lebanese University, Beirut, Lebanon; ^3^Lebanese University Nutrition Surveillance Center (LUNSC), Lebanese Food Drugs and Chemical Administrations, Lebanese University, Beirut, Lebanon; ^4^Hariri School of Nursing, American University of Beirut, Beirut, Lebanon; ^5^World Health Organization Regional Office for the Eastern Mediterranean, Cairo, Egypt; ^6^Al Hadi Laboratory and IVF Center, Beirut, Lebanon; ^7^Department of Computer Science, Al-Quds University, Jerusalem, Palestine; ^8^Department of Computer Engineering, Istinye University, Istanbul, Turkey; ^9^Department of Medical Laboratory Sciences, Al-Quds University, Jerusalem, Palestine; ^10^Department of Biology, College of Science, University of Bahrain, Zallaq, Bahrain; ^11^Department of Obstetrics and Gynecology, King Hamad University Hospital, Busaiteen, Bahrain; ^12^Department of Obstetrics and Gynecology, College of Medicine and Medical Sciences, Arabian Gulf University, Manama, Bahrain; ^13^Department of Clinical Nutrition, Faculty of Applied Medical Sciences, Taibah University, Madinah, Saudi Arabia; ^14^National Nutrition Committee (NNC), Saudi Food and Drug Authority (Saudi FDA), Riyadh, Saudi Arabia; ^15^Department of Nutrition and Food Technology, Al-Huson University College, Al-Balqa Applied University, As-Salt, Jordan; ^16^Department of Human Nutrition, College of Health Sciences, Qatar University, Doha, Qatar

**Keywords:** pregnant women, COVID-19, maternal nutrition, USDA recommendations, adherence

## Abstract

During pregnancy, woman’s diet is one of the most preeminent factors affecting mother and child’s health. Prior to the coronavirus disease-19 (COVID-19) pandemic, inadequate maternal diet and low adherence to dietary guidelines was reported among pregnant women in the Arab countries. Nowadays, COVID-19 infection during pregnancy is widely discussed among literature. However, there is limited data on the health impacts of the COVID-19 pandemic on non-infected pregnant women. This substantially larger group also suffered significant lifestyle changes during the lockdown period. The aim of the study is to characterize dietary patterns, intake and adherence to the United States Department of Agriculture (USDA) pregnancy guidelines before and during the COVID-19 pandemic in Arab pregnant women. Using a specially designed questionnaire and using the snowball sampling method, the survey was carried out among a convenient sample of 1,939 pregnant women from five Arab countries. Our study found an increment in the consumption of cereals, fruits, vegetables, dairy products, meats, and nuts that occurred during the pandemic compared to the preceding period. Despite this noticeable increase during the pandemic, the Arab pregnant women in this study had significantly lower adherence to the USDA pregnancy guidelines. The daily consumption of almost all food groups was lower than the USDA’s daily recommendations, except for fruits intake, which was higher than the daily standard. Demonstrated poor adherence to prenatal USDA dietary guidelines by Arab pregnant women can lead to numerous deficiencies and health risks among their offspring. In conclusion, our study showed that before and during the COVID-19 pandemic, poor adherence to dietary recommendations occurred in a considerable number of Arab pregnant women. The findings emphasize the need for nutritional education and intervention during prenatal visits.

## Introduction

By March 21, 2020, the novel 2019 coronavirus disease (COVID-19) had infected over 292,000 confirmed cases worldwide, with 18,000 confirmed cases in the Eastern Mediterranean countries (1). Because of the new COVID-19’s extremely contagious nature, numerous governments have taken exceptional measures to prevent disease transmission, such as suspending public transportation and restricting the whole social life (2). These measures affected the lifestyles of many people, including pregnant women, in a significant way. In the time of pandemics, mother’s nutritional patterns become of high-priority for the mother’s and child’s health (2). Healthy eating habits and adherences to evidenced-based guidelines is one of the requirements for a successful pregnancy (3). Poor adherence to dietary standards and guidelines can lead to nutritional deficits that affects the pregnancy’s progress and the child’s healthy growth (2, 3). During crises, good dietary patterns, combined with adequate intake, increases the likelihood of an optimal pregnancy outcomes (2, 4). During pregnancy, the development of maternal tissues, fetal growth, and breast milk production increase nutritional requirements (4). Different national and international organizations advocate dietary improvement, such as adhering to the USDA pregnancy guidelines which show the amount of food recommended for pregnant women including fruits, vegetables, grains, dairy, and protein foods (5). Despite evidence supporting the importance of maternal nutrition, various studies reveal that few women follow adequate diets (6). A study conducted in Jordan showed low adherence to dietary guidelines among 99% of pregnant women during the pre-COVID-19 time (6). This study was conducted to provide a situational analysis with regards to maternal nutrition and to assess the adherence to the USDA’s guidelines among pregnant women in five Arab countries (Lebanon, Palestine, Jordan, Saudi Arabia, and Bahrain). Due to unavailability of common Eastern Mediterranean guidelines, USDA guidelines were adopted in this study to determine cutoffs of serving size consumption, which may not fully reflect the situation of Eastern Mediterranean women. The selection of countries was based on a collaborative work between researchers from these countries. Despite the availability of nutrition awareness information, the working hypothesis anticipated in this study that most pregnant women ignore the appropriate advice and do not adhere to the USDA dietary guidelines.

## Materials and Methods

### Questionnaire

A cross-sectional study, using the snowball sampling method, was conducted during the COVID-19 pandemic in five Arab countries (Lebanon, Palestine, Jordan, Saudi Arabia and Bahrain). A web-based questionnaire was disseminated through social media websites (available at the link https://www.palnut.org/frontend/web/index.php?r=survey/survey/index) to collect data from pregnant women. The questionnaire used in our survey was previously validated in two published data (6, 7). The current survey investigated pregnant women’s sociodemographic characteristics, maternal medical history, eating patterns, food consumption, physical activity patterns, anthropometric data, smoking, anxiety, and depression. The first section of the questionnaire inquired about pregnancy and its progression. It includes questions about the mother’s age, health status, and diseases. Moreover, the socio-demographic characteristics included education, residency, and economic situation. The second section questioned about the daily serving sizes from each food group (bread, pasta, cereal, vegetables, fruits, meat, poultry, fish, nuts, sweets, fast food, fats, and oils) consumed during the day and week prior to completing this survey. Participants were also asked about their mental health (anxiety and depression) and smoking habits, as well as any physical activities they engaged in. In the current study, we did not cover the pre-pregnancy period, but rather the period preceding the COVID-19 pandemic and the pandemic period only. The questionnaire had various questions, some with only one option for each topic and others with open-ended answers.

### Variables and Measurements

#### Body Mass Index

The pregnant women’s pre-pregnancy body mass index (BMI) was calculated according to the World Health Organization (WHO)’s instructions (8).

#### Depression

Depression was assessed using the validated Patient Health Questionnaire (PHQ-9). It was selected according to its effectiveness in identifying depression (9). This questionnaire encompasses nine depression-related issues. Depression levels were classified as: no depression = 0–4, mild = 5–9, moderate = 10–14, moderately severe = 15–19, and severe = >20.

#### Anxiety

The clinically validated tool entitled “seven-item Generalized Anxiety Disorder-7 (GAD-7)” was used to assess anxiety symptoms (10). Respondents rank items on a four-point scale ranging from 0 (never) to 3 (nearly every day). Anxiety severity was defined by total scores of 0–4 for no anxiety, 5–9 for mild, 10–14 for moderate, and 15 or higher for severe anxiety (10).

#### Physical Activity

Pregnant woman was considered active if she claimed that she engaged in any degree of physical activity (low, moderate, or high) for at least half an hour per day (11).

#### Dietary Guideline for Pregnant Women

Recommended amounts of food were classified based on the USDA’s guideline for pregnant women (12). The food group’s consumption was dichotomized based on USDA guidelines’ cutoff points (5). Each food group was assigned a score 0 or 1, with 0 indicating lower intake than the USDA’s daily recommendations and 1 indicating higher intake.

For the food group bread, rice, and other cereals, less than six servings indicate a lower intake, and greater than or equal to six servings indicate a higher intake. A lower intake of fruit is equal to less than two servings, while a higher intake is greater than or equal to two servings. Less than 2.5 servings of vegetables suggest a lower intake, while 2.5 servings or more indicate a higher intake. Less than 5.5 servings in the protein food group indicates a lower intake, while greater than or equal to 5.5 servings indicates a higher intake. As for the dairy food group, a lower intake is equal to three servings, while a higher intake is greater than or equal to three servings.

Additionally, the adherence score to the USDA guidelines was derived by adding the adherence to recommendations for each food group. This variable was then dichotomized into two categories: low adherence score (0–2) and high adherence score (3–5).

### Inclusion/Exclusion Criteria

The following criteria conditioned the data collection: (i) pregnancy since the pre-COVID-19 pandemic period; (ii) pregnancy of normal course; (iii) the woman’s age > 18; (iv) place of residence—the listed five countries; (v) replying to all questions; (vi) consenting participating in the study. Moreover, the exclusion criteria were conception during the intra-COVID-19 pandemic period and some risk factors such as miscarriage.

### Ethical Consideration

The study design obtained written approval of the Ethics Committee in Scientific Research of Lebanese University (CUER#30-2020), as well as universities from all participating countries. Before completing the questionnaire, each participant was informed of the study’s goal and ensured the confidentiality of their information. The completion of the questionnaire was voluntary and anonymous. Consenting to participate in the study was considered as a necessary component.

### Statistics and Data Analysis

Continuous variables were expressed as means and standard deviations (SDs), while categorical variables were shown as frequencies and percentages. Chi-square test was used to compare variables among the five countries, while ANOVA test was applied for continuous variables. The Paired sample *t*-test was used to compare continuous variables before and during the pandemic, while the McNemar test (a marginal homogeneity test for paired data) was used to compare categorical variables. The statistical significance level was set at *p*-value < 0.05, and the statistical analysis was carried out using IBM Corp. Released 2017. IBM SPSS Statistics for Windows, Version 25.0. Armonk, NY: IBM Corp.

## Results

A total number of 1,939 women participated in the current survey. Table 1 shows the sociodemographic characteristics of the study participants. The respondents’ mean (±SD) age was 28.5 (±5.4) years. Highest age was reported among Saudi pregnant women [29.6 (±5.7)] while the youngest pregnant women were from Palestine [27.7 (±5.5)]. Similarly, the vast majority (77%) were young adults, with Saudi Arabia having the highest proportion (83%). Palestine, on the other hand, had the highest number of youth (32%) (*p*-value < 0.001). Around two-thirds (62%) of the participants had received a bachelor’s or graduate’s degree, with Saudi Arabia having the highest percentage (81%) and Jordan having the lowest (53%). Lebanon, however, was among the best in terms of graduate degrees (28%). Only 38% of women worked, with Bahrein having the highest rate (49%) and Saudi Arabia having the lowest (31%) (*p*-value < 0.001). Furthermore, Saudi Arabia had the greatest proportion of unemployed pregnant women (69%). Most pregnant women (64%) reported a drop in household income, with Jordan having the greatest rate (84%) and Bahrain having the lowest (36%). Participants from Lebanon also reported the largest income loss, with a 9% decrease.

**TABLE 1:** Socio-economic characteristics of the study participants, by country.

Variable	Jordan *n* = 531 *n* (%)	Palestine *n* = 609 *n* (%)	Lebanon *n* = 363 *n* (%)	Saudi Arabia *n* = 256 *n* (%)	Bahrain *n* = 180 *n* (%)	*P*-value
**Age (Year: mean ± SD)**	28.8 ± 5.5	27.7 ± 5.5	28.3 ± 4.8	29.6 ± 5.7	29.2 ± 4.9	<0.001
Youth (18–24)	128 (24.1)	192 (31.6)	91 (25.1)	43 (16.8)	33 (18.3)	
Young adults (>25)	403 (75.9)	416 (68.3)	272 (74.9)	213 (83.2)	147 (81.7)	
**Education level**						<0.001
Less than high school	33 (6.2)	49 (8.0)	11 (3.0)	2 (0.8)	16 (8.9)	
High school diploma	107 (20.2)	125 (20.5)	28 (7.7)	20 (7.8)	36 (20.0)	
Diploma	109 (20.5)	71 (11.7)	82 (22.6)	25 (9.8)	19 (10.6)	
Bachelor’s degree	250 (47.1)	319 (52.4)	142 (39.1)	175 (68.4)	95 (52.8)	
Graduate degree	32 (6.0)	45 (7.4)	100 (27.5)	34 (13.3)	14 (7.8)	
**Employment status**						<0.001
Employed	204 (38.4)	194 (31.9)	169 (46.6)	79 (30.9)	89 (49.4)	
Unemployed	327 (61.6)	414 (68.0)	194 (53.4)	177 (69.1)	91 (50.6)	
**Family income**						<0.001
Decreased	446 (84.0)	434 (71.3)	181 (49.9)	111 (43.4)	64 (35.6)	
Increased	8 (1.5)	26 (4.3)	33 (9.1)	12 (4.7)	15 (8.3)	
No change	77 (14.5)	149 (24.5)	149 (41.0)	133 (52.0)	101 (56.1)	

The health characteristics of the respondents are summarized in Table 2. The mean value of the pre-pregnancy BMI for respondents was 25.1 (±8) kg/m^2^, with more than half having normal BMI (55%), 28.4% being overweight, 12% being obese (class I, II, and III), and the remaining (5%) being underweight. Bahrain had the highest pre-pregnancy BMI 27.5 (±12.1) and Palestine had the lowest 24.5 (±7.2). Furthermore, Bahrein had the highest proportions of overweight and obese (52%) and Palestine the lowest (34%) (*p*-value < 0.001). COVID-19 was diagnosed in only a small percentage of the respondents (7%). Saudi Arabia had the greatest percentage of infection (14%) while Palestine had the lowest (3%) (*p*-value < 0.001). Around 40% of pregnant women said they had health complications, with Jordan placing first (72%) and Bahrein last (43%). Most people stated they did not have diabetes (97%) or gestational diabetes (95%) or hypertension (98%) or thyroid disorders (96%). Nearly a third of the individuals (31%) suffered from moderate to severe depression, with Jordan topping the list (53%) and Bahrein trailing behind (33%) (*p*-value 0.001). Furthermore, roughly 16% were anxious, with Palestine having the highest rate (19%) and Bahrein having the lowest (7%). Around two-thirds of the participants (64%) were physically active, with Jordan having the most (77%) and Saudi Arabia having the least (64%) active persons (46%) (*p*-value < 0.001). Last but not least, more than a quarter of pregnant women (27%) smoked during their pregnancy, with Jordan having the highest percentage (67%) and Bahrein having the lowest (8%) (*p*-value < 0.001).

**TABLE 2:** Health characteristics of the study participants, by country.

Variable	Jordan	Palestine	Lebanon	Saudi Arabia	Bahrain	*P*-value
	*n* = 531	*n* = 609	*n* = 363	*n* = 256	*n* = 180	
	*n* (%)	*n* (%)	*n* (%)	*n* (%)	*n* (%)	
**Pre-pregnancy BMI (mean ± SD) kg/m^2^**	25.2 ± 6.0	24.5 ± 7.2	24.7 ± 9.7	24.9 ± 5.8	27.5 ± 12.1	< *0*.001
**Pre-pregnancy BMI categories**						< *0*.001
Normal	272 (54.1)	313 (60.2)	173 (58.6)	104 (49.5)	72 (44.7)	
Underweight	14 (2.8)	30 (5.8)	17 (5.7)	16 (7.6)	6 (3.7)	
Overweight	176 (34.9)	127 (24.4)	69 (23.4)	62 (29.5)	45 (27.9)	
Obese class I	32 (6.4)	39 (7.5)	28 (9.5)	18 (8.6)	24 (14.9)	
Obese class II	6 (1.2)	7 (1.4)	5 (1.7)	9 (4.3)	7 (4.4)	
Obese class III	3 (0.6)	4 (0.8)	3 (1.0)	1 (0.5)	7 (4.4)	
**Diagnosed with COVID-19**						< *0*.001
No	479 (90.2)	591 (97.1)	350 (96.4)	220 (85.9)	159 (88.3)	
Yes	52 (9.8)	18 (2.9)	13 (3.6)	36 (14.1)	21 (11.7)	
**Health problems**						< *0*.001
No	148 (27.87)	205 (33.66)	148 (40.8)	111 (43.4)	103 (57.2)	
Yes	383 (72.13)	404 (66.3)	215 (59.2)	145 (56.6)	77 (42.8)	
**Diabetes**						0.005
No	509 (95.9)	598 (98.2)	358 (98.6)	248 (96.9)	169 (93.9)	
Yes	22 (4.1)	11 (1.8)	5 (1.4)	8 (3.1)	11 (6.1)	
**Gestational diabetes**						< *0*.001
No	504 (94.9)	590 (96.9)	353 (97.3)	247 (96.5)	138 (76.7)	
Yes	27 (5.1)	19 (3.1)	10 (2.7)	9 (3.5)	42 (23.3)	
**Hypertension**						0.003
No	511 (96.2)	598 (98.2)	362 (99.7)	253 (98.8)	178 (98.9)	
Yes	20 (3.8)	11 (1.8)	1 (0.3)	3 (1.2)	2 (1.1)	0.908
**Thyroid disorders**						
No	512 (96.4)	581 (95.4)	347 (95.6)	244 (95.3)	172 (95.6)	
Yes	19 (3.6)	28 (4.6)	16 (4.4)	12 (4.7)	8 (4.4)	
**Depression status**						< *0*.001
No depression	27 (9.2)	31 (12.6)	19 (13.6)	14 (16.5)	26 (26.3)	
Mild	110 (37.5)	102 (41.3)	72 (51.4)	34 (40.0)	40 (40.4)	
Moderate	91 (31.1)	63 (25.5)	25 (17.9)	24 (28.2)	20 (20.2)	
Moderately severe	46 (15.7)	35 (14.2)	17 (12.1)	11 (12.9)	11 (11.1)	
Severe	19 (6.5)	16 (6.5)	7 (5.0)	2 (2.4)	2 (2.0)	
**Anxiety status**						0.004
No anxiety	172 (42.9)	77 (31.3)	63 (44.4)	32 (37.6)	50 (50.0)	
Mild	156 (38.9)	122 (49.6)	63 (44.4)	45 (52.9)	43 (43.0)	
Moderate	64 (16.0)	38 (15.4)	14 (9.9)	7 (8.2)	7 (7.0)	
Severe anxiety	9 (2.2)	9 (3.7)	2 (1.4)	1 (1.2)	0 (0)	
**Physical activity**						< *0*.001
Inactive	439 (36.0)	108 (23.3)	144 (38.3)	74 (45.1)	60 (54.5)	53 (50.5)
Active	780 (64.0)	356 (76.7)	232 (61.7)	90 (54.9)	50 (45.5)	52 (49.5)
**Smoking status during pregnancy**						< *0*.001
No	493 (72.6)	31 (33.3)	166 (73.1)	116 (71.6)	107 (90.7)	73 (92.4)
Yes	186 (27.4)	62 (66.7)	61 (26.9)	46 (28.4)	11 (9.3)	6 (7.6)

### Daily Intake of the Main Food Groups

Table 3 shows the dietary intake among pregnant women from the five countries before and during the pandemic.

**TABLE 3:** Number of servings consumed per day and adherence to United States Department of Agriculture (USDA) recommendations of the major food groups consumed by pregnant women before and during the coronavirus disease-19 (COVID-19) pandemic, by country.

	Number of servings (mean ± SD) and percentage of adherence to USDA before the COVID-19 pandemic						Number of servings (mean ± SD) and percentage of adherence to USDA during the COVID-19 pandemic						
Food groups	Jordan	Palestine	Lebanon	Saudi Arabia	Bahrain	*p*-value	Jordan	Palestine	Lebanon	Saudi Arabia	Bahrain	*p*-value	*p*-value^#^
Bread, rice and other cereals (*N* = 593)	6.9 ± 6.7	5.1 ± 4.7	5.0 ± 6.5	3.1 ± 2.3	5.3 ± 4.0	< *0*.001	8.2 ± 8.4	5.8 ± 5.2	5.3 ± 7.5	3.9 ± 3.2	5.4 ± 4.4	< *0*.001	< *0*.001
Bread, rice and other cereals						< *0*.001						< *0*.001	< *0*.001
<6 servings	68 (50.4)	180 (67.7)	59 (70.2)	48 (85.7)	37 (56.1)		49 (36.3)	145 (55.8)	59 (69.4)	44 (77.2)	35 (54.7)		
≥6 servings	67 (49.6)	86 (32.3)	25 (29.8)	8 (14.3)	29 (43.9)		86 (63.7)	115 (44.2)	26 (30.6)	13 (22.8)	29 (45.3)		
Fruits (*N* = 543)	2.8 ± 2.9	2.4 ± 1.9	2.2 ± 1.6	1.4 ± 1.0	2.0 ± 1.3	0.002	3.0 ± 2.4	2.5 ± 2.0	2.3 ± 2.0	1.8 ± 1.2	2.3 ± 1.5	0.001	< *0*.001
Fruits						0.001						0.001	< *0*.001
<2 servings	59 (36.4)	74 (35.2)	34 (43.6)	32 (66.7)	25 (43.9)		37 (23.4)	62 (29.2)	29 (37.2)	27 (55.1)	20 (35.1)		
≥2 servings	103 (63.6)	136 (64.8)	44 (56.4)	16 (33.3)	32 (56.1)		121 (76.6)	150 (70.8)	49 (62.8)	22 (44.9)	37 (64.9)		
Vegetables (*N* = 566)	2.6 ± 2.4	2.4 ± 2.5	2.2 ± 1.7	1.6 ± 1.2	1.8 ± 1.4	0.014	3.1 ± 2.9	2.6 ± 2.6	2.3 ± 2.1	1.8 ± 1.3	2.3 ± 2.0	0.008	< *0*.001
Vegetables						0.005						< *0*.001	< *0*.001
< *2*.5 servings	96 (63.2)	178 (73.6)	54 (72.0)	46 (86.8)	45 (81.8)		75 (50.3)	173 (71.2)	56 (74.7)	43 (82.7)	38 (69.1)		
≥2.5 servings	56 (36.8)	64 (26.4)	21 (28.0)	7 (13.2)	10 (18.2)		74 (49.7)	70 (28.8)	19 (25.3)	9 (17.3)	17 (30.9)		
Milk and milk products (*N* = 559)	2.4 ± 2.4	1.8 ± 1.8	1.6 ± 1.4	1.7 ± 1.6	2.0 ± 1.9	0.015	2.9 ± 2.6	2.1 ± 2.2	1.5 ± 1.3	1.9 ± 1.8	2.1 ± 1.9	< *0*.001	< *0*.001
Milk and milk products						0.002						< *0*.001	< *0*.001
<3 servings	127 (71.3)	182 (83.5)	65 (90.3)	41 (87.2)	44 (75.9)		98 (56.6)	173 (77.6)	68 (90.7)	37 (78.7)	43 (74.1)		
≥3 servings	51 (28.7)	36 (16.5)	7 (9.7)	6 (12.8)	14 (24.1)		75 (43.3)	50 (22.4)	7 (9.3)	10 (21.3)	15 (25.9)		
White and red meats and nuts (*N* = 565)	5.26 ± 3.7	4.5 ± 5.6	4.2 ± 4.6	3.3 ± 4.1	3.8 ± 2.2	0.011	5.8 ± 3.6	4.9 ± 6.2	3.7 ± 3.2	3.4 ± 2.5	4.6 ± 5.6	0.001	0.001
White and red meats and nuts						< *0*.001						< *0*.001	< *0*.001
< *5*.5 servings	166 (57.6)	124 (80.0)	55 (79.7)	40 (93.0)	40 (78.4)		133 (48.8)	116 (79.5)	55 (83.3)	35 (83.3)	38 (71.7)		
≥5.5 servings	121 (42.2)	31 (20.0)	14 (20.3)	3 (7.0)	11 (21.6)		142 (51.6)	30 (20.5)	11 (16.7)	7 (16.7)	15 (28.3)		
Adherence to food groups (score)						0.559						0.082	< *0*.001
No/Low (0–2)	274 (83.8)	240 (83.9)	83 (86.5)	54 (91.5)	55 (82.1)		263 (80.9)	231 (81.9)	86 (89.6)	52 (88.1)	49 (74.2)		
Moderate/High (3–5)	53 (16.2)	46 (16.1)	13 (13.5)	5 (8.5)	12 (17.9)		62 (19.1)	51 (18.1)	10 (10.4)	7 (11.9)	17 (25.8)		

*p < 0.001.*

*^#^Comparison between pre-COVID-19 and intra-COVID-19 periods.*

The consumption of cereals, fruits, vegetables, dairy products and protein group increased significantly during the pandemic compared to the period before (*p*-value < 0.001; Table 3). Additionally, before and during the pandemic, women’s daily consumption of almost all food groups was lower than the USDA’s daily recommendations, with the exception of fruit consumption, which was higher than the daily standard.

#### Bread, Rice and Other Cereals Group

Prior to the pandemic, in the five countries, more than half of women consumed less than six servings of breads, rice and other cereals. However, it was shown that the number of servings consumed per day of breads, rice and other cereals increased significantly during the pandemic (*p*-value < 0.001). For instance, around 64% of women in Jordan, 44.2% in Palestine, 30.66% in Lebanon, 22.8% in Saudi Arabia and 45.3% in Bahrain consumed more than six servings of this food group per day during the pandemic (*p*-value < 0.001). The lowest number of servings consumed was observed in Saudi Arabia (3.1 ± 2.3 versus 3.9 ± 3.2) and the highest was in Jordan (6.5 ± 6.7 versus 8.2 ± 8.4) (*p*-value < 0.001).

#### Fruits Group

Before the pandemic, more than 60% of pregnant women living in Jordan (63.6%) and Palestine (64.8%) and more than half of women living in Lebanon (56.4%) and Bahrain (56.1%) were adhering to the USDA’s recommendations related to fruits intake (≥2 servings per day). This was not the case of Saudi pregnant women who consumed less than two servings per day of fruits before the pandemic. Nevertheless, an increase in the consumption of fruits was remarkable during the pandemic (*p* < 0.001). Before and during the pandemic, the lowest number of fruits servings was observed among Saudi women (1.4 ± 1 versus 1.8 ± 1.2). On the other hand, Jordanian women ranked first in consuming fruits before and during the pandemic (2.8 ± 2.9 versus 3 ± 2.4) (*p*-value < 0.001).

#### Vegetables Group

Prior to the pandemic, most of pregnant women in the five countries showed poor adherence to USDA’s recommendations with regards to vegetable intake (≥2.5 servings per day). Nevertheless, the percentage of women showing acceptable adherence to USDA recommendations increased during the pandemic in all countries except in Lebanon (decrease of 3%) (*p*-value < 0.001). The lowest percentage of vegetable consumption was observed among Saudi women of which 86.8 and 82.7% consumed less than 2.5 servings per day, before and during the pandemic, respectively (*p*-value < 0.001). Otherwise, in both study periods, the highest percentage of pregnant women who consume vegetables was observed in Jordan (Table 3).

#### Milk and Dairy Products Group

Before the COVID-19 pandemic, around three quarter of pregnant women in all countries showed poor consumption of milk and dairy products (<3 servings per day). The lowest consumption was seen among Lebanese pregnant women of which only 9% adhered to the USDA recommendations. During the pandemic, the number of milk and dairy product’s servings increased significantly in all countries, except in Lebanon (*p*-value < 0.001).

#### White, Red Meats, and Nuts Group

Prior to the pandemic, around half the pregnant women in Jordan, 80% in Palestine, 80% in Lebanon, 93% in Saudi Arabia, and 79% in Bahrein consumed less than 5.5 servings of white or red meats or nuts. The percentage of adherence to the USDA recommendations for meats and nuts group increased significantly during the pandemic except in Palestine and Lebanon (*p*-value < 0.001).

### Adherence to the United States Department of Agriculture Recommendations Among Countries

According to Table 3, 83.8% of women in Jordan, 83.9% in Palestine, 86.5% of women in Lebanon, 91.5% in Saudi Arabia, and 82.1% in Bahrein showed poor adherence to the USDA’s recommendations before the COVID-19 pandemic. Nevertheless, the percentage of adherence increased significantly during the pandemic (*p*-value = 0.001). It increased of 3% in Jordan, 2% in Palestine, 3.4% in Saudi Arabia and 8% in Bahrein. However, it decreased of 3.1% in Lebanon (*p*-value < 0.001).

## Discussion

This study describes the food consumption and adherence to the USDA’s guidelines among pregnant women in five Arab countries. The food consumption in the pre-COVID-19 period as well as during the pandemic was unfavorable regarding almost all food groups. Notably, indecorous dietary patterns and poor adherence to the USDA recommendations (in more than 80% of pregnant women) was obvious. This finding came hand in hand with data reported by Tayyem et al. (6) where only 1.1% of pregnant women adhered to dietary guidelines in the pre-COVID-19 time (6).

### Comparison With International Studies

Otherwise, our findings concerning the increase in the consumption of all food groups were concordant with the findings of a longitudinal Chinese study which found that the consumption of vegetables, fruits, dairy products, and cereals was significantly higher among pregnant women during the pandemic (13). Furthermore, Hillier et al. revealed a substantial increase in the consumption of fruit and vegetable along with a decrease in egg, fried fast foods, coffee and tea consumption from pre-pregnancy period to during pregnancy in the pre-COVID-19 time (14).

Starchy carbohydrates and fiber containing whole grain cereals and vegetables are the fundamental of a healthy diet. According to the USDA guidelines, the intake of 18–24 g of fiber during the second and third trimester allow for good body’s functioning (5). Due to its richness in minerals, vitamins and dietary fibers, pregnant women are recommended to include wholegrain cereal products in their daily diet. Although the primary source of energy and nutrients should be derived from this food group and should be part of each main meal, 65% of the women in this study consume less than six portions per day from this food group.

A large number of antioxidants (vitamin A, C, and E, carotenoids and flavonoids) are derived from the inclusion of vegetables and fruits in pregnant women’s diets. They provide also folates, potassium and fiber. According to the USDA’s recommendations, an amount of 300 and 350 g per day in the first semester, and in the second and third trimester—300 g and 450 per day of fruits and vegetables, respectively, should be included in pregnant women’s diet (5). Similarly, according to WHO, the intake of vegetables and fruit in the diet of pregnant women should be of high-priority (15). In this study, women mostly consumed fruits in a way higher than the daily standard. In comparison with data from pre-COVID-19-time, our findings came hand by hand with the results obtained in the study conducted by Dere’n et al. (16), where the majority of the women consumed fruit between meals, and one third outstretched for sweets during pregnancy (16). Another study conducted by Kobiołka et al. (17) showed that, between meals, fruit was the most preferred snack consumed by pregnant women (17).

According to many international dietary standards, protein intake should be increased throughout pregnancy, particularly in the second and third trimesters. Referring to the USDA guidelines, 165–195 g (second and third trimester) of protein sources allow for a proper functioning of the body (5). Protein is required for the tissue and placenta of both the mother and the fetus (18). Primary sources of protein could be derived from animal sources (red, lean meat and its products, skimmed milk and its products, fish and poultry). Thus, the daily intake should increase of 1, 8, and 26 g in the first trimester, the second trimester, and in the third trimester of pregnancy (19). Our findings revealed that a significant proportion of respondents consumed poorly this food group (70% in pre-COVID-19 period compared to 65% in the intra-COVID-19 period). Our results are tied well with the literature where Abd-Elmohdy Emara (20) investigated that 37, 31, and 27% of pregnant women consumed white meat 3–4 times per week, 2–3 times per week, and at least once per day, respectively. Furthermore, according to the same study, 43, 12, and 33% of women ate red meat 3–4 times per week, 2–3 times per week, and occasionally, respectively (20).

Moreover, during pregnancy, the body’s demand for iron rises, and red meats are a rich source of iron (21). Because of their increased vitamin B_12_ and iron content, pregnant women should have white and red meats in their weekly diet. Our findings indicated that a significant proportion of respondents consumed poorly this food group (70% in pre-COVID-19 period compared to 65% in the intra-COVID-19 period), which may result in anemia in the short term and low birth weight in the long term (22). Our results backs up findings from a systematic analysis by Caut et al. (23), which found that in 91 and 55% of included studies, pregnant women were not adhering to iron and calcium dietary recommendations, respectively (23).

Dairy products, rich in protein, riboflavin and calcium, are essential for pregnant women, alongside bread, vegetables, fruits, and meat (24). According to WHO, a dietary intake of 1,200 mg/day of calcium for pregnant women is recommended (15). To meet this need, a pregnant woman should drink daily three cups of skimmed milk (5). Fermented milk drinks, rich in nutritious protein, vitamin B_2_, and calcium are widely recommended in the diets of pregnant women because they provided probiotic bacteria (3). According to our findings, pregnant women’s diets have a low percentage of milk and dairy products. In our study, 80% of the subjects were drinking milk and fermented milk drinks less than three servings per day. The research of Kobus-Cisowska et al. (25) and Suliga (26) yielded similar results (25, 26). Likewise, 43% of pregnant women in the study by Dere’n et al. (16), consumed dairy products once a day, and 48% more often (16). Regular physical activity during pregnancy, known to be beneficial to both physical and mental health, in addition to a balanced diet, has an impact on the mother’s and child’s short and long-term health (27–29). Nevertheless, most pregnant women in our study (64%) show usually high levels of physical activity. According to WHO, at least 150 min of moderate intensity physical activity per week is recommended for adults (30). Unless there are medical restrictions, regular and moderate exercise is recommended for pregnant women (27–29). Due to the need to adapt the physiological and psychological changes during pregnancy, pregnant women may struggle with the sedentary behavior and become physically inactive (3). Finally, despite the well-known health benefits related to practicing regular physical activity during pregnancy, over 40% of women do not adhere to the recommendations.

### Comparison With Arab Countries’ Data

To our knowledge, no data was published among Arab countries that investigate the consumption patterns of pregnant women amid the COVID-19 pandemic. Thus, our findings were compared to many data published in which the food consumption was reported for the whole population. In Lebanon, the findings of a national study aligned our findings in which a significant increase in the number of meals consumed per day during the pandemic compared to before the pandemic (all *p* < 0.001) was observed (31). However, there was a significant decrease in physical activity engagement during the lockdown compared to before the pandemic (all *p* < 0.001) (31). Another study in Lebanon showed that home isolation due to COVID-19 induced an increase in the consumption of legumes and pulses (3.2%, *p*-value = 0.001) and whole wheat groups (2.8%, *p*-value = 0.03). In contrast, a decrease of 5.4, 6.9, 5.8, 5.1, 3.1, 3.4, and 2.8% was observed in the consumption of fruits (*p*-value = 0), vegetables (*p*-value = 0), processed meats, poultry, and fish (*p*-value = 0) and other dairy products (*p*-value = 0), respectively. In Lebanon, since the ordeals of COVID-19, economic crisis, and the Beirut port explosions, food insecurity became an immediate problem for households in Lebanon. Between November 2020 and March 2021, 9 in every 16 households ate less than two meals per day and more than 70% of them skipped their meals to spare food. This explains the difference in term of food consumption among Lebanese pregnant women compared to other countries (32). At the Arab countries level, a recent study conducted in 10 countries including Jordan, Palestine, Lebanon, Saudi Arabia and Bahrain showed that, before and during the pandemic, most food groups were consumed less or equal to four times per week which indicated a poor dietary diversity among the countries’ population (33). This finding along with our findings describe the nutritional situation and food consumption patterns among pregnant women on one side and the whole population on the other side.

### Limitations

This study has some limitations. First, this study was conducted online through convenience sampling that could probably lead to skewed sample characteristics in some countries. Second, respondents were asked to recall the food categories’ consumption patterns prior to lockdown, which could have caused recall bias. Third, although this sampling method does not always guarantee the generalizability of the results, it can remain an effective method for estimating the likelihood of potential relationships between variables (34–36). Finally, although it is necessary that all Arab countries adhere to evidenced-based guidelines, multiple factors can limit guidelines’ adherence, including income, food availability and affordability, individual beliefs and preferences, cultural traditions, and educational, social, geographical, and environmental aspects.

## Conclusion

Although it is clear that most Arab pregnant women ameliorated their food consumption patterns amid the COVID-19 pandemic, however, the food consumption in the pre-COVID-19 period as well as during the pandemic was unfavorable. Obviously, most Arab pregnant women showed low adherence to the USDA pregnancy recommendations. Thus, prenatal nutrition education and intervention are required. More research is needed to uncover modifiable variables and dietary concerns in pregnant women.

## Data Availability Statement

The original contributions presented in the study are included in the article/supplementary material, further inquiries can be directed to the corresponding authors.

## Ethics Statement

The studies involving human participants were reviewed and approved by Lebanese University. The patients/participants provided their written informed consent to participate inthis study.

## Author Contributions

MH and RT: conceptualization, data curation, formal analysis, investigation, methodology, project administration, supervision, validation, and writing—original draft preparation. RH: data curation, methodology, writing—original draft preparation, and writing—review and editing. AA-J, CF, MH, RA, and MI: methodology and writing—review and editing. MA, SO, RQ, SA, KB, JA, and NA-B: data curation, methodology, and writing—review and editing. All authors have read and agreed to the published version of the manuscript.

## Author Disclaimer

The views expressed in this article and they do not necessarily represent the views, decisions or policies of WHO, Saudi FDA or the other institutions with which the authors are affiliated.

## Conflict of Interest

The authors declare that the research was conducted in the absence of any commercial or financial relationships that could be construed as a potential conflict of interest.

## Publisher’s Note

All claims expressed in this article are solely those of the authors and do not necessarily represent those of their affiliated organizations, or those of the publisher, the editors and the reviewers. Any product that may be evaluated in this article, or claim that may be made by its manufacturer, is not guaranteed or endorsed by the publisher.
